# Dynamic conformational switching underlies TFIIH function in transcription and DNA repair and impacts genetic diseases

**DOI:** 10.1038/s41467-023-38416-6

**Published:** 2023-05-13

**Authors:** Jina Yu, Chunli Yan, Thomas Dodd, Chi-Lin Tsai, John A. Tainer, Susan E. Tsutakawa, Ivaylo Ivanov

**Affiliations:** 1 Department of Chemistry, Georgia State University, Atlanta, GA USA; 2 Center for Diagnostics and Therapeutics, Georgia State University, Atlanta, GA USA; 3 Department of Molecular and Cellular Oncology, The University of Texas MD Anderson Cancer Center, Houston, TX USA; 4 Molecular Biophysics and Integrated Bioimaging, Lawrence Berkeley National Laboratory, Berkeley, CA USA

**Keywords:** Nucleotide excision repair, Computational biophysics

## Abstract

Transcription factor IIH (TFIIH) is a protein assembly essential for transcription initiation and nucleotide excision repair (NER). Yet, understanding of the conformational switching underpinning these diverse TFIIH functions remains fragmentary. TFIIH mechanisms critically depend on two translocase subunits, XPB and XPD. To unravel their functions and regulation, we build cryo-EM based TFIIH models in transcription- and NER-competent states. Using simulations and graph-theoretical analysis methods, we reveal TFIIH’s global motions, define TFIIH partitioning into dynamic communities and show how TFIIH reshapes itself and self-regulates depending on functional context. Our study uncovers an internal regulatory mechanism that switches XPB and XPD activities making them mutually exclusive between NER and transcription initiation. By sequentially coordinating the XPB and XPD DNA-unwinding activities, the switch ensures precise DNA incision in NER. Mapping TFIIH disease mutations onto network models reveals clustering into distinct mechanistic classes, affecting translocase functions, protein interactions and interface dynamics.

## Abstract (web summary)

The study unveils the structure, dynamics and regulatory mechanisms of the TFIIH protein assembly underpinning its divergent functions in gene expression and genome maintenance. Models link positions of TFIIH mutations to genetic disease phenotypes.

## Introduction

Nucleotide excision repair (NER) is a biochemical pathway vital for genome integrity. NER stands out among all DNA repair pathways for its versatility and precision in removing the widest array of structurally unrelated DNA lesions caused by ultraviolet radiation, reactive oxygen species, environmental mutagens, and chemotherapeutic agents^1–3^. The pathway can be conceptually subdivided into four steps: (1) damage recognition; (2) DNA unwinding and damage verification; (3) dual incision of the lesion-containing DNA strand; and (4) repair synthesis to fill the resultant DNA gap. Adding to the complexity, NER features two sub-pathways, global genome NER (GG-NER) and transcription-coupled NER (TC-NER), that differ only in the damage recognition step. TC-NER^4–6^ is essential for lesion removal from the template strand during transcription and is activated by the recruitment of Cockayne Syndrome B protein (CSB) to lesion-arrested RNA polymerase II (Pol II)^7–12^. By contrast, in GG-NER a heterotrimeric complex of XPC, HR23B and Centrin 2 (CETN2)^13^ detects the lesion and, in a twist-to-open mechanism, melts the DNA duplex at the damaged site^14–19^. Afterward, GG-NER and TC-NER converge to recruit a host of repair factors, including transcription factor IIH (TFIIH)^20–31^, which is the centerpiece of the NER machinery. Assisted by XPA^13,32–34^, TFIIH unwinds DNA^35^ around the lesion and its XPD subunit^36,37^ performs damage verification^38–41^ by scanning the damage-containing single-stranded DNA (ssDNA). Replication protein A (RPA)^32,42^ binds and protects the newly unwound ssDNA on the undamaged strand. Two nucleases, XPG and XPF/ERCC1^43–48^, are recruited by TFIIH/XPA, forming a pre-incision complex (PInC), which is critical for ensuring only licensed DNA incisions occur. XPF and XPG complete the DNA cleavage on both sides of the lesion, precisely excising the damaged DNA segment as an oligonucleotide of 26 or 27 nucleotides^49^. At the same time, Polδ, RFC, and PCNA are loaded onto the DNA on the 5′ side of the lesion and start DNA synthesis to replace the excised region. Finally, TFIIH and XPG depart, and DNA ligase completes repair by sealing the nicked DNA. Clearly, TFIIH is key for the assembly, coordination, and regulation of the intricate NER machinery. Yet, many aspects of its tightly orchestrated functions remain unknown at the level of structure and dynamics.

TFIIH is a large (460 kDa) and dynamic protein assembly that serves multiple functions in GG-NER, TC-NER and transcription initiation^30^. TFIIH encompasses ten subunits, including seven core subunits (XPB, XPD, p62, p52, p44, p34, p8) and three CDK-activating kinase (CAK) subunits (CDK7, Cyclin-H, MAT1)^50^. Key to TFIIH’s remarkable functional versatility is its modular architecture, which allows it to adapt to diverse protein partners. Recent cryo-EM studies reveal the dramatic structural rearrangements that TFIIH undergoes from its apo state (apo-TFIIH) to the transcription preinitiation complex (holo-PIC)^20,21^ and to the GG-NER complex (NER-TFIIH)^32^. Yet, our understanding of the complex conformational switching that underpins TFIIH’s diverse functions remains fragmentary.

In this study, we carry out detailed comparative analyses of apo-TFIIH, holo-PIC and NER-TFIIH in terms of structure and dynamics. Starting from the cryo-EM map of the NER-TFIIH complex, we added missing regions, including the C- and N-terminal ends of XPA and the p62 subunit of TFIIH. Our previous work afforded suitably complete structural models of apo-TFIIH and holo-PIC^51^. The three cryo-EM based atomic models served as starting points for extensive microsecond-long molecular dynamics (MD) simulations of the TFIIH assembly in all three functional states. We also used chain-of-replicas path optimization methods to model DNA translocation by TFIIH’s two ATPase subunits, XPB and XPD. Global motions relevant for translocation were analyzed separately for the XPB and XPD molecular motors and then compared to the global dynamics of these modules within the NER-TFIIH assembly. Next, we employed difference contact network analysis (dCNA) over the apo-TFIIH, holo-PIC and NER-TFIIH conformational ensembles. Remarkably, from this analysis we find that XPB and XPD coordinate their activities during NER to enable precise DNA incision. Thus, our study sheds light on how TFIIH dynamically reshapes itself and self-regulates depending on functional context.

NER is also exceptional for the variety of clinical manifestations associated with its genetic impairment^1,52–54^. Defects in NER provide a paradigm for the diverse clinical consequences of DNA damage and are associated with severe human genetic diseases^55–60^—ultraviolet radiation–sensitive syndrome (UVSS); xeroderma pigmentosum (XP) characterized with extreme cancer predisposition; trichothiodystrophy (TTD) and Cockayne syndrome (CS) leading to progressive neurodevelopmental defects, recurrent infections, and high mortality at a young age.

This striking clinical heterogeneity is incompletely understood at the level of structure and biological mechanisms. In this respect, the observed dramatic structural changes from apo-TFIIH to holo-PIC and NER-TFIIH translate into equally significant differences in functional dynamics, differentially affecting allosteric communication through these complexes. In turn, knowledge of the distinct dynamics from MD simulations has aided us in interpreting the effects of patient mutations associated with XP, XP/TTD, TTD, and XP/CS disease phenotypes. Our results provocatively suggest that disease mutations could be differentiated by phenotype if one considers not only their structural aspects but also their ability to disrupt critical nodes in the protein dynamic network. Thus, our study unveils the critical structural and dynamic characteristics that define TFIIH’s function in GG-NER versus transcription initiation and the allosteric mechanisms acting in cognate DNA recognition and processing.

## Results

### Reconstruction of a complete NER-TFIIH complex and comparison to apo-TFIIH and PIC

**Fig. 1:**
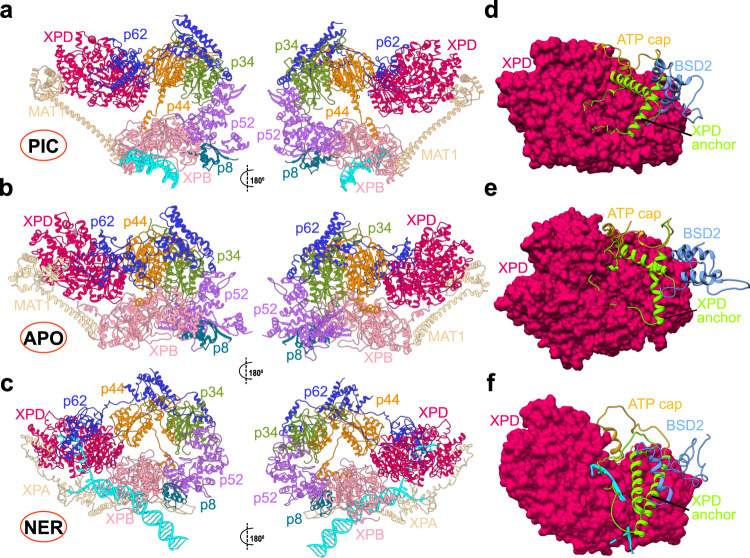
Cryo-EM based models of TFIIH in apo state, holo-PIC and NER-TFIIH reveal how this multifaceted assembly reorganizes with changed functional context. Anterior and posterior view of TFIIH in **a** the holo-PIC assembly; **b** apo-TFIIH; and **c** the NER lesion scanning complex. XPD anchor (light green), BSD2 (light blue) and ATP-cap (gold) elements of p62 positioned on the XPD surface in **d** PIC-TFIIH; **e** apo-TFIIH; and **f** NER-TFIIH. The TFIIH subunits are shown in cartoon representation colored as follows: XPD dark red, p62 blue, p44 orange, p34 green, p52 purple, p8 dark cyan, XPB pink; MAT1 and XPA are shown in tan and DNA is cyan.

The TFIIH/XPA/DNA cryo-EM structure^32^ was pivotal for offering an unprecedented molecular view of the NER protein machinery and for explaining the functional role of core TFIIH subunits during the lesion scanning stage of NER. While the core TFIIH subunits could be modeled with confidence into the cryo-EM density, certain flexible structural elements, including the C- and N-terminal ends of XPA and the entire p62 subunit, remained unmodeled. Inclusion of these regions is essential for the success of molecular simulations aimed at elucidating the functional dynamics of the GG-NER assembly. To produce a suitably complete model, the missing regions were traced in the original EM density and built de novo. The entire assembly was then flexibly fitted into the cryo-EM density (EMD-4970). Comparison of the resultant NER-TFIIH model to our previous models of apo-TFIIH and holo-PIC is shown in Fig. 1 and provides unanticipated mechanistic insights. Newly modeled regions are shown in Supplementary Fig. 1.

In the PIC, TFIIH encompasses all ten subunits whereas the NER complex includes only the seven core subunits^28^. Two ATPase subunits, XPB and XPD, are adjacent in our NER-TFIIH model but spaced out in the holo-PIC model. Apo-TFIIH features an intermediate XPB–XPD spacing. The four middle subunits (p8, p52, p34, p44) lie in a characteristic horseshoe shape, more widely open in the PIC and narrower in the NER model. The newly modeled p62 (Fig. 1d–f) is the most extended of the core TFIIH subunits, traversing and interlacing the surfaces of p34, p44 and XPD. Its N-terminal half dramatically reorients (Fig. 1d–f) from apo-TFIIH to the DNA-bound PIC and NER complexes. This observation carries key functional implications. In the PIC, the XPD anchor domain of p62 inserts into XPD’s DNA-binding groove to inhibit its ssDNA translocase activity (Fig. 1d)^51^. In NER, XPD’s activity is essential and, correspondingly, our NER model reveals that p62 shifts away from XPD’s DNA-binding cleft to allow the passage of ssDNA (Fig. 1f). Thus, instead of blocking XPD, the rearranged p62 now caps the channel through which ssDNA is threaded. This conformational switching involves complete repositioning of the p62’s BSD2 and XPD anchor domains (Fig. 1d–f; Supplementary Movie 1).

Another critical difference between the models is the positioning of the MAT1 subunit. In the PIC, MAT1 establishes the spacing from the XPB DNA-damage recognition (DRD) domain to the XPD Arch domain via an 86-Å long α-helix and a helical bundle. MAT1 also bridges Cyclin-H and CDK7 to form TFIIH’s kinase subcomplex (CAK), which is key for transcription regulation^30,51^. In the PIC, TFIIE, p62 and MAT1 are principally responsible for the interface between core-PIC and TFIIH and, through their interactions, duplex DNA is directed away from XPD and toward the RNA polymerase cleft upon exiting XPB (Fig. 1a–c). This arrangement enables the formation of a nascent transcription bubble^51^. By contrast, in the NER-TFIIH assembly TFIIE is not present, while MAT1 and CAK are displaced by XPA and by XPD-ssDNA binding. These changes result in a dramatically altered DNA path through the NER complex (Fig. 1a–c) with DNA directed toward XPD. Surprisingly, similar to MAT1, XPA spans both XPB and XPD but, unlike MAT1, brings them closer together. The newly modeled N-terminal region of XPA features mostly unstructured loops except for one helix anchored on XPD (Fig. 1a–c). The unstructured NTE of XPA cannot space XPB and XPD apart. The C-terminal end of XPA consists of a long helix that serves as a clamp on duplex DNA, preventing its dissociation from XPB. The helical clamp ends with an antiparallel β-sheet, which firmly anchors the XPA N-terminus to p8 (Fig. 1a–c). XPA also provides a β-hairpin to separate the DNA strands: one of the strands passes through XPD while the other exits the complex near the XPA zinc-finger domain.

### Global motions underpinning the distinct DNA translocation mechanisms of XPB and XPD

DNA unwinding in NER critically depends on the activities of TFIIH’s translocase subunits, XPB and XPD^30,36,37^. Yet, the nature of their cooperation in opening DNA and lesion scanning during NER remains enigmatic. XPB and XPD both have DNA helicase and ATP hydrolysis activities independently from TFIIH^35,39,61–63^. Thus, we first address the question of how XPB and XPD act on DNA by modeling the isolated XPB and XPD chains. To unravel the respective mechanisms, we rely on chain-of-replicas path optimization methods. We used the partial nudged elastic band method (PNEB)^64^ to compute minimum free energy paths (MFEP) for DNA translocation through XPB and XPD, respectively. Each path represents the entire translocation mechanism, including intermediate states in the ATP hydrolysis cycle. Notably, the MFEPs also identify the dominant global motions of XPB and XPD that cause forward movement on DNA in response to changing nucleotide state.

**Fig. 2:**
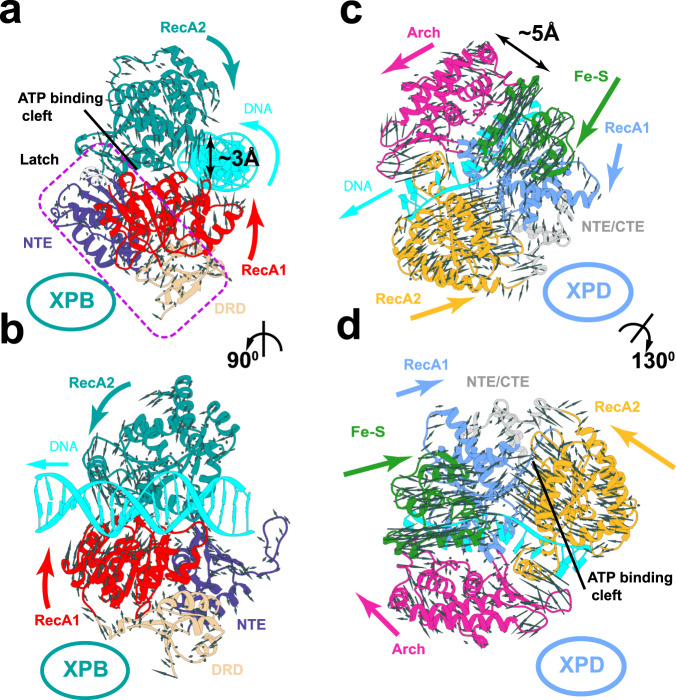
Chain-of-replicas path optimization methods reveal the DNA translocation mechanisms of XPB and XPD. Global motions (gray arrows) of the XPB domains in **a** top and **b** side orientation. RecA1 is shown in red, RecA2 in dark cyan, NTE in purple, DRD in tan, dsDNA in cyan. XPB latch is indicated by violet dashed outline; opening/closure of XPB ATPase domain cleft is indicated by a black double arrow. Global motions (gray arrows) of the XPD domains in **c** top and **d** side orientation. The Arch domain is shown in magenta, RecA1 in light blue, RecA2 in gold, Fe–S in green, NTE-CTE in gray, ssDNA in cyan. Opening/closure of the Arch and Fe–S domains is indicated by a black double arrow.

XPB accommodates duplex DNA in the groove created by its two ATPase domains (denoted RecA1 and RecA2). In the MFEP, RecA1 and RecA2 rotate in opposite directions, conveying the overall rotational motion onto the DNA duplex (Supplementary Movie 2). While the RecA2 rotational axis roughly coincides with the DNA axis, the RecA1 rotational axis is shifted in a way that promotes forward movement of the DNA duplex along the length of the XPB groove. Overall, translocation proceeds in one base-pair/cycle increments and involves forward shifting and rotation of the DNA duplex accompanied by ~3-Å opening and closing of the tandem ATPase domains (Fig. 2a, b). While XPB’s NTE and DRD domains participate in this collective dynamics, their motions are subdued compared to RecA1 and RecA2 (Fig. 2a, b). Instead, NTE and DRD form a latch that braces the XPB ATPase core from the opposite side of the DNA-binding groove. The latch reinforces the interface between the ATPase domains, which would otherwise be held together by a single flexible linker. In TFIIH the latch is incorporated into a larger collar structure comprised of NTE, DRD, p52 XPB-binding domain and p8. This arrangement imparts directionality to the motions of the ATPase core and assists forward DNA translocation.

By contrast, XPD translocates on ssDNA and its global dynamics involves the mutual displacements of four structural domains – RecA1, RecA2, iron sulfur domain (Fe–S) and Arch domain. ssDNA is slotted through a narrow channel formed by the four domains, which is lined with positive residues for electrostatic complementarity to the phosphodiester backbone. Reptation of ssDNA through the channel is facilitated by the nucleotide-induced closure of the tandem ATPase domains and the concomitant ~5-Å opening of the spacing between the Arch and Fe–S domains (Fig. 2c, d; Supplementary Movie 3). ssDNA encounters two constricted regions along its translocation path (Supplementary Fig. 2). The first constriction is located between the Arch and Fe–S domains and involves residues Y211, Y192, R196, R122, R380, H135, H304, L220, F196, R299 (Supplementary Fig. 2). Passage of ssDNA through this region is gated by the opening and closing motions of the Arch and Fe–S during the ATP hydrolysis cycle (Supplementary Movie 3). The gating motion is functionally significant as blocking of ssDNA inside XPD between the Arch and Fe–S domains could signal the presence of a bulky lesion. The second constriction lies on the opposite side of the XPD DNA-binding cleft. This part of the XPD channel is tight, highly complementary to ssDNA in terms of shape and electrostatics and is lined with positively charged (R511, R683, K689, K507, R686) or aromatic residues (F508, Y627, Y625) (Supplementary Fig. 2). Unlike the first constriction, this constriction is not transient and does not fall apart at any point during the ATP hydrolysis cycle. Nucleotides pass through this region one at a time facilitated by stacking and unstacking motions of the complementary aromatic residues (Supplementary Movie 3).

### TFIIH conformational dynamics is predicated on functional context

**Fig. 3:**
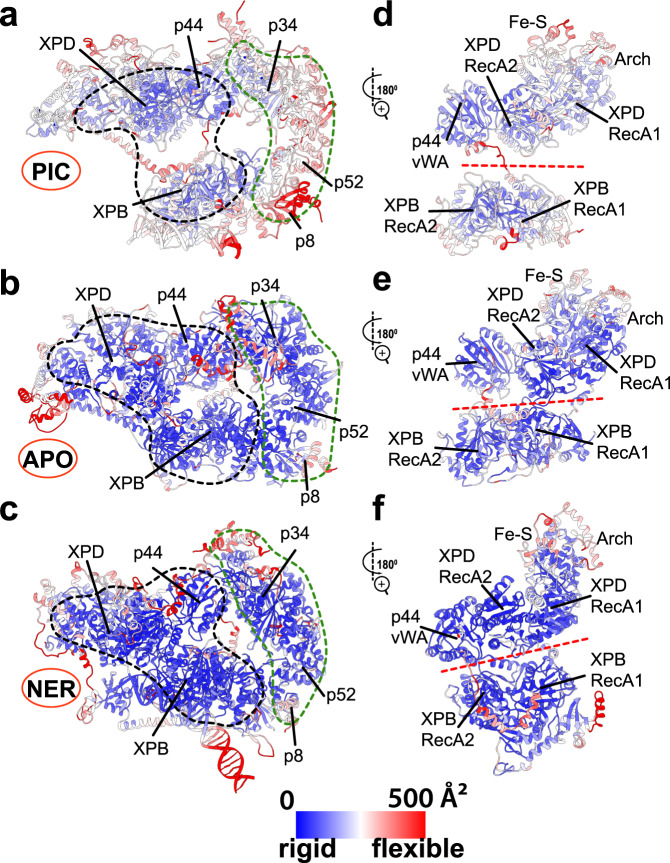
TFIIH dramatically alters its flexibility and dynamics depending on functional context. Computed B-factors mapped onto the structural models of **a** PIC-TFIIH, **b** apo-TFIIH and **c** NER-TFIIH. B-factor values are colored from low (blue) to high (red). Close-up views of the rigid (blue) versus flexible (red) structural elements at the XPB–XPD interface in **d** PIC-TFIIH, **e** apo-TFIIH and **f** NER-TFIIH. Black dashed outline highlights an unexpected rigid anchor region formed by p44/XPB and part of XPD in the TFIIH-NER complex. The interface of XPB and XPD is highlighted by a red dashed line. The TFIIH lever arm (p8, p52, p34) is indicated by a green dashed outline.

To unravel the precise functional roles of XPB and XPD within the TFIIH assembly, we performed extensive molecular dynamics simulations (~1-μs/system) of apo-TFIIH, NER-TFIIH and holo-PIC. We first assess the relative rigidity/flexibility of the numerous TFIIH structural elements and link the observed differences among the three models to their putative functional roles. To this end, we mapped the computed B-factors from each simulation onto the three TFIIH structural models (Fig. 3). Despite their common overall architecture, the apo-TFIIH, NER-TFIIH and holo-PIC exhibit drastically different dynamics. Overall flexibility appears to be closely tied to the observed XPB–XPD spacing. PIC-TFIIH is the most flexible assembly and, correspondingly, features the largest spacing. With no appreciable interface between XPB and XPD (Fig. 3d), the lever arm of TFIIH (p8, p52 and p34) is free to swing toward the RNA polymerase cleft, amplifying the motion of the XPB molecular motor. This motion directs dsDNA toward Pol II and assists the formation of the nascent transcription bubble. By contrast, the most rigid segment of TFIIH includes the core of XPD (apart from the mobile Fe–S domain), p62 and the p44 subunit (Fig. 3a). The XPD, p62 and p44 subunits participate in a larger ridge of structural stability that extends across the Pol II–TFIIE–TFIIH interface and anchors the XPB molecular motor to the rest of the initiation machinery. In this context, both XPD/p44 rigidity and the lever arm flexibility are key for TFIIH function in transcription initiation. Apo-TFIIH features an intermediate XPB–XPD spacing and a nascent interface between XPD and RecA1 of XPB (Fig. 3e). This results in much decreased mobility of the TFIIH lever arm compared to PIC (Fig. 3b). Decreased B-factors are seen for both XPB and XPD, but notably RecA1 and RecA2 of XPB remain dynamically independent. Since the XPD-XPB interface affects only one of the XPB RecA domains, the second domain is free to swing and mediate XPB-driven dsDNA translocation. Thus, in apo-TFIIH both XPB and XPD retain their translocase activities despite decreased overall mobility. The NER-TFIIH complex is the most rigid of the three assemblies (Fig. 3c). Rotation of XPB and XPD further reduces the spacing between the ATPase subunits. In this arrangement, both RecA modules of XPB are stably bound to XPD. P44, XPB and XPD strengthen their interfaces resulting in a rigid structural block (Fig. 3f). Importantly, the XPD Arch and Fe–S domains can still open and close, allowing translocation of ssDNA through the XPD cleft (Supplementary Movie 4).

### Network analysis uncovers a key regulatory switch controlling XPB/XPD activities in NER

The size, complexity, and flexibility of TFIIH complicate analysis of its functional dynamics. To dissect this staggering complexity, we apply graph-theoretical approaches that map dynamic information from MD onto graphs representing the protein topology (nodes are the protein residues; edges connect contacting residues). Graph edges are assigned weights based on contact probabilities derived from MD. We then use the Girvan-Newman algorithm to partition the network into dynamic communities, which constitute the independently moving structural elements of TFIIH. To systematically compare the three TFIIH functional states, we take advantage of difference contact network analysis (dCNA)^65^. First, we compute residue contact networks from each conformational ensemble—apo-TFIIH, PIC-TFIIH and NER-TFIIH. Second, we construct a consensus network wherein edges denote stable contacts across all three ensembles. Third, we identify the dynamic communities and map them onto the consensus network. Finally, we subtract the contact probabilities of the individual networks, yielding difference contact network graphs. dCNA maps information from multiple MD ensembles onto a single consensus community structure, allowing us to discover which community interfaces are gaining or losing contacts upon switching between any two functional states.

**Fig. 4:**
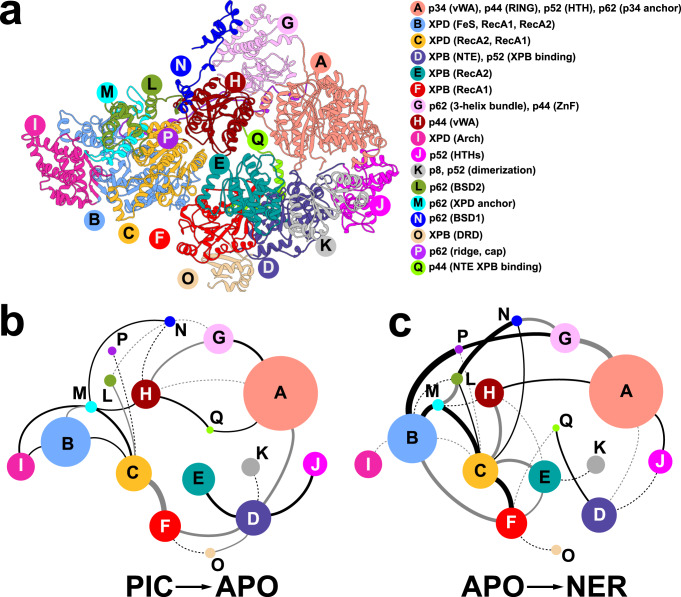
Community networks underlying changes in TFIIH dynamics in transcription versus NER. **a** Consensus communities are identified from difference contact network analysis and mapped to the structure of apo-TFIIH. Communities are color-coded and labeled. Net change in contact probabilities across TFIIH communities during **b** the PIC to apo transition and **c** the apo to NER transition. The radius of each community vertex denotes the size (number of residues) of the community. The gray and black edges between communities indicate overall gain(+)/loss(-) of dynamic contacts.

**Fig. 5:**
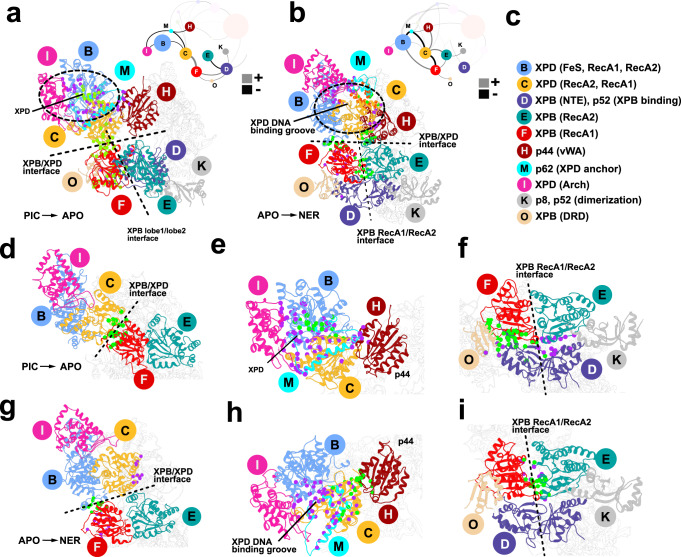
Network analysis reveals conformational switching at TFIIH community interfaces. Residues experiencing the largest gain/loss of contact probability during the PIC to apo and apo to NER transitions are mapped onto the TFIIH structure and shown as green or purple dots, respectively. Dynamic communities of XPB, XPD and adjacent subunits are colored as in Fig. 4. TFIIH lever arm communities are shown in gray. dCNA subgraphs for these communities are shown as insets, with gray or black edges indicating gain(+)/loss(-) of dynamic contacts Contact changes are shown for the following transitions: **a** PIC to apo; **b** apo to NER. **c** community identities in the consensus network are indicated; labels denote the principal domains/structural elements belonging to a community. **d**–**f**, Close-up views of interfaces with the largest contacts gain (green)/loss (purple) during the PIC to apo transition: **d** the XPB–XPD interface; **e** XPD-p44 and XPD-p62 interfaces; **f** the XPB ATPase domains and the collar region. Close-up views of interfaces with the largest contacts gain (green)/loss (purple) during the apo to NER transition: **g** the XPB–XPD interface; **h** the XPD-p44 and XPD-p62 interfaces; **i** the XPB ATPase domains and the collar region.

Our analysis identifies 17 distinct TFIIH communities (Fig. 4), which represent the smallest dynamically independent modules observed in all three simulation ensembles. Aggregate contact probability changes between communities for the PIC → apo and apo → NER conformational transitions are shown in Fig. 4, giving a coarse-grain view of TFIIH structural reorganization. In Fig. 5, we identify residues experiencing the largest gain or loss of contact probability and map these onto the TFIIH structure. This representation gives a more fine-grain depiction of conformational switching at TFIIH community interfaces.

First, we focus on the two ATPase subunits. XPB encompasses four dynamic communities (Fig. 4a), including three that separate strictly along domain boundaries: community F (RecA1); community E (RecA2); and community O (DRD). The fourth community (D) spans the XPB NTE domain and the p52 XPB-binding domain, which move together as a single module. By contrast, XPD partitioning does not follow domain structure (Fig. 4a). Community B is the largest among the XPD-associated communities and incorporates most of the RecA1 domain, a smaller segment of RecA2 and the entire Fe–S domain. Conversely, community C contains the larger segment of RecA2 along with the remaining part of RecA1. Community I principally coincides with the Arch domain.

During the PIC → apo transition, we observe a large gain in contact probability between communities C and F, which parallels the formation of the nascent XPD-RecA1 interface of apo-TFIIH (Figs. 4b and 5d). Notably, there is no net change in contact probability between communities E and F (Figs. 4b and 5d), suggesting that the XPB ATPase modules remain dynamically independent and capable of dsDNA translocation both in apo-TFIIH and the PIC. Dynamic changes are also observed in communities D (NTE, p52), O (DRD) and K (p8, p52), which wrap around the XPB ATPase core as a collar and modulate its translocase activity.

By contrast, in the apo → NER transition we observe complete reorganization of the XPB–XPD interfaces, resulting in the loss of dynamic independence of the XPB ATPase domains (Fig. 4c). Specifically, mutual rotation and shift of XPB and XPD cause the nascent interface (C–F) to be completely abolished and replaced with two tighter interfaces between communities C–E and B–F (Figs. 4c and 5b, g). P44 (community H) also gains substantial interactions with communities C and E forming the most rigid core of the NER-TFIIH complex (Fig. 4c; Fig. 5h). Residues at the newly formed XPD/XPB, XPD-p44 interfaces are highly conserved (Supplementary Fig. 3). Importantly, the RecA1 and RecA2 modules of XPB gain interfacial contacts and move together as a single dynamic module (Fig. 5b, i). XPD, on the other hand, experiences little change in interaction strength between communities B, C, and I (Fig. 4c) and remains capable of ssDNA translocation (Supplementary Movie 4). Thus, in the NER-TFIIH complex XPB is inactivated while XPD is active and poised to scan ssDNA for lesions.

The structural reorganization of p62 is also notable. In transcription initiation, p62 plays a key regulatory role by inserting into the XPD DNA-binding groove and inactivating the translocase. In the PIC → apo transition, p62 contacts are reorganized, relieving the XPD inhibition. Correspondingly, community M (p62 XPD anchor) loses contacts (Figs. 4b and 5e) with the XPD ATPase core and the Arch domain (community I). This frees the XPD-RecA1 and RecA2 modules to move independently as seen by the contact probability loss between communities B and C. In the apo → NER transition, we observe further loss of contacts (Figs. 4c and 5h) between community P (p62 ridge) and community B (RecA1) suggesting a much looser association of p62 with XPD in the NER complex. This added p62 flexibility is likely functionally significant as it is needed for XPD recruitment to the expanding NER bubble in the early steps of the pathway^13^.

**Fig. 6:**
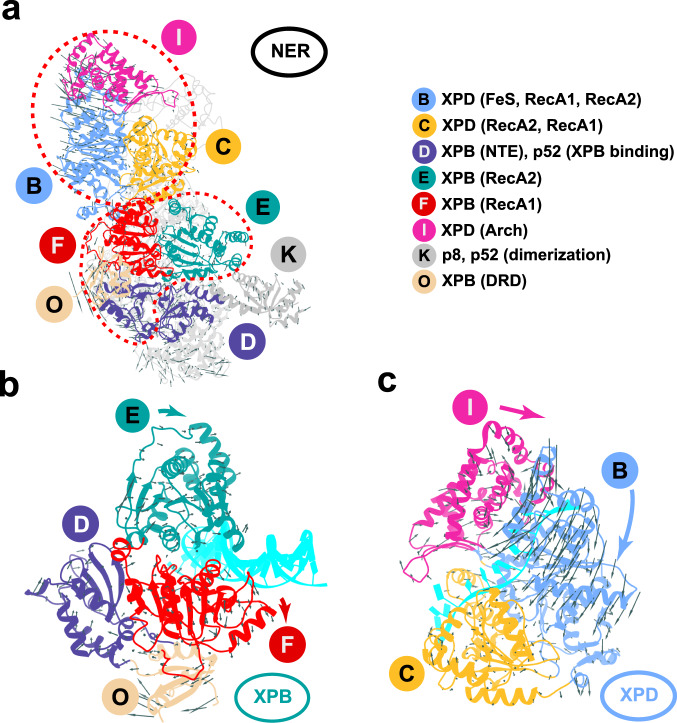
Mapping of the global motions of the NER-TFIIH complex onto the dCNA communities shows that translocation dynamics is enabled for XPD and suppressed for XPB during lesion scanning. **a** The first principal mode from PCA analysis of the NER-TFIIH complex; gray arrows indicate the directionality of the motions of the Cα atoms of the assembly; **b** Zoomed view showing the motion of the XPB communities; **c** Zoomed view showing the motion of XPD communities. Communities are colored the same as in Fig. 4.

We further verify our observations from dCNA using principal component analysis (PCA)^66^. PCA is a dimensionality reduction technique that projects the conformers from the MD simulation trajectories into a space defined by a few lowest principal modes obtained by diagonalizing the covariance matrix. The PCA modes recapitulate the largest amplitude global motions of TFIIH, which are often the most functionally relevant. PCA suitably complements dCNA, as it allows us to visualize not only the partitioning of TFIIH into dynamic modules but also the principal directions in which the modules are moving. Thus, we map the global motions of the NER-TFIIH complex onto the dCNA communities (Fig. 6; Supplementary Movie 4). We confirm that in the NER-TFIIH complex the XPB RecA1 and RecA2 domains cannot open up and displace to cause dsDNA translocation. Instead, the XPB ATPase core acts as a single dynamic module, which executes a slight rocking motion around dsDNA (Fig. 6b, Supplementary Movie 4). The dynamics of the TFIIH lever arm subunits is also suppressed. By contrast, the XPD subunit is the sole region of TFIIH-NER that exhibits large-scale dynamics. Specifically, the DNA-binding groove of XPD can still open between the Arch and the Fe–S domain, and the directionality of the opening motion closely resembles the dynamics of free XPD on ssDNA (Fig. 6c, Supplementary Movie 4).

Thus, we conclude that in the context of the PIC XPD is inactive while XPB is active. Conversely, in the NER-TFIIH lesion scanning complex XPB is inactive while XPD is active. In apo-TFIIH both XPD and XPB activities are possible depending on the cognate DNA substrate. Collectively, we uncover a remarkable TFIIH internal regulatory mechanism that switches XPB and XPD activities making them mutually exclusive in GG-NER and in transcription initiation.

### Disease mutations disrupt key community interfaces impacting TFIIH structure and dynamics

**Fig. 7:**
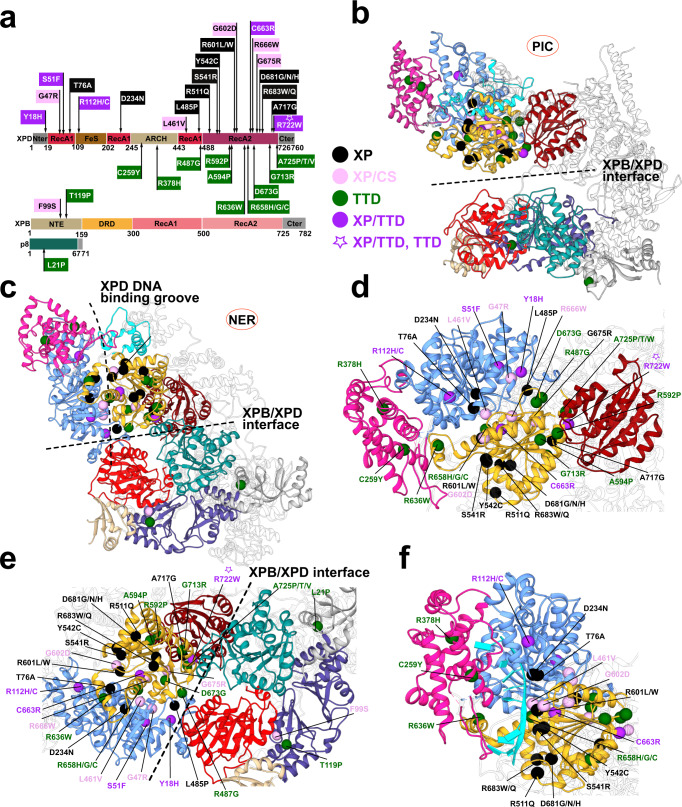
Human disease mutations mapped onto TFIIH show distinct patterns within protein-protein and community interfaces. **a** TTD, XP, XP/CS and XP/TTD point mutations mapped onto XPD, XPB, and p8 subunits do not co-localize by disease on primary sequence. **b** Map of human disease mutations (spheres) onto the PIC-TFIIH structure shown in cartoon representation and colored by community. **c** Map of human disease mutations (spheres) onto the NER-TFIIH structure shown in cartoon representation and colored by community. **d** Zoomed view of mutations within XPD. **e** Zoomed view of mutations at the XPB–XPD interface. **f** Zoomed view of mutations lining the path of ssDNA in XPD.

TFIIH is an intricate molecular machine with diverse functions in GG-NER, TC-NER and transcription initiation. Genetic mutations that impair these vital pathways cause distinct autosomal recessive genetic disorders—xeroderma pigmentosum, trichothiodystrophy, and xeroderma pigmentosum/Cockayne syndrome^55–57,67–69^. The fact that XP, TTD, XP/CS and XP/TTD are recessive disorders complicates analyses that seek to correlate structural changes upon mutation to disease severity. In patients who are compound heterozygotes^70^ both alleles may contribute to the observed phenotype and could differentially affect the function of TFIIH in NER and transcription. In turn, this could partially account for the broad range of clinical heterogeneity. Our dynamic network models incorporate data from multiple MD ensembles and account for TFIIH’s dramatic structural reorganization. To link TFIIH structure and dynamics to disease phenotypes, we mapped 34 known missense disease mutations of TFIIH (Fig. 7) onto our network models. Disease mutations are irregularly dispersed throughout XPB, XPD and p8, highlighting the functional importance of the two translocase subunits^39,56^. In Supplementary Data 1, we annotate disease mutations by phenotype and discuss the mutation-induced structural changes in TFIIH. Instances where contributions from the second allele may influence phenotype are also described. An example is the XPD R722W mutation, observed in two patients, both compound heterozygotes. The second allele mutations, which were different in each patient gave rise to two distinct phenotypes—XP/TTD for the patient carrying S51F mutation, and TTD for the patient carrying R378H mutation.

We had previously assessed TFIIH community structure only for the holo-PIC complex, which is key for transcription initiation^51^. Remarkably, we observe that most disease mutations line up along community boundaries and that transcription deficient TTD mutants mapped to key interfaces of the two translocases with adjacent TFIIH subunits: XPB with p44, p8 or p52; or XPD with MAT1, p44, and p62. We are now able to refine our analysis and classification of disease mutations by comparing the dynamic communities between the PIC and the NER-TFIIH network models (Supplementary Fig. 4; Supplementary Data 1). The most striking difference between the models concerns XPD community subdivision. Notably, the RecA1, RecA2 and Fe–S domains are all dynamically coupled in the PIC, forming a single community that also includes segments of p62 (Fig. 7b, Supplementary Fig. 4, and Supplementary Movie 5). By contrast, in the GG-NER complex RecA1 and RecA2 become dynamically independent, and a new boundary emerges between community B (mostly RecA1 and Fe–S) and community C (mostly RecA2) (Fig. 7c, Supplementary Fig. 4, and Supplementary Movie 5). The B–C community interface is especially rich in disease mutations. Mutations previously classified as internal in the PIC community model now become interfacial and significant for the functional dynamics of the GG-NER model. Other mutants previously assigned to the XPD-p44 community interface now lie at the interfaces of three dynamic communities (B–C, B–H and C–H) (Fig. 7c, d, Supplementary Fig. 4 and Supplementary Movie 5). Importantly, side by side comparison of community structure in the PIC and GG-NER functional states allows far better insight into the distinct positioning of disease mutations by phenotype.

Pure XP mutations occur mainly along the ssDNA path through XPD (e.g., T76A, D234N, S541R, Y542C, R511Q, R683W/Q, R601L/W) but occupy only the half of the DNA-binding groove proximal to the DNA junction (Fig. 7f and Supplementary Movie 5). The distal part of the groove between Fe–S and Arch domains remains free of XP mutations. This observation highlights the relative importance of the two constrictions along the ssDNA path in XPD: the distal constriction is transient whereas the proximal is key for ssDNA translocation along the surface of RecA2 and confers most of the affinity of XPD for ssDNA.

XP/CS mutations also occur only in XPD (e.g., G47R, L461V, G602D, R666W, G675R) and are distributed along the B-C community boundary following a line roughly perpendicular to the XPD DNA-binding groove (Fig. 7d, f and Supplementary Movie 5). The line passes through some of the most critical and dynamically important regions of the translocase — the ATP-binding site and the key helicase motifs of XPD — and extends toward the functionally significant XPD-p44 boundary. They are also in proximity to p62, which inhibits XPD in transcription. Disrupting these regions would impact ATP hydrolysis, the mutual displacement of RecA1 and RecA2 needed for NER and the XPD-p44 interface integrity, which is required for both NER and transcription initiation. Predictably, XP/CS mutations are deficient in transcription, TC-NER and GG-NER, resulting in the most severe disease phenotype.

By contrast, TTD mutants are located on the periphery of the XPD’s ATPase core (Fig. 7b, c; and Supplementary Movie 5), mapping to important interfaces of XPD with p44 (communities C and H), MAT1 (community I) and p62. The TTD phenotype is manifested in two sets of clinical features that reflect the dual functionality of TFIIH in transcription and DNA repair. First, TTD mutations may cause in vivo instability of TFIIH, resulting in the characteristic brittle hair/nails and scaly skin of TTD patients^71^. Similar instability of other (non-repair) proteins needed for gene expression can cause the same features^72,73^. By contrast, the progressive premature aging features of repair-deficient TTD patients can be attributed to defects in TC-NER compounded by deficient GG-NER. When GG-NER is severely impacted, XP features may co-occur.

We analyzed the effect of mutations on protein stability using the Rosetta ddG protocol^74,75^. The obtained ddG scores can be interpreted as a proxy for the effect of the mutation on thermodynamic stability. Larger positive values suggest significant protein destabilization upon mutation. Conversely, small values have smaller effect on thermodynamic stability but may have significant impact on dynamics and function. TTD mutations, especially mutants at the XPD/p44 interface exhibited larger ddG scores (Supplementary Fig. 5**;** Supplementary Data 2) and, thus, are predicted to decrease TFIIH stability and be partially deficient in transcription. We posit that TTD mutations weaken assembly of TFIIH subunits while retaining residual XPB translocase activity, which is essential for transcription.

Disease mutations can also affect XPB function. Intriguingly, none of the XPB mutations localize to RecA1 or RecA2 (Fig. 7e). The XPB ATPase activity is indispensable for transcription and its complete abolishment likely prevents cell viability. Instead, missense mutations (F99S (XP/CS) or T119P (TTD) from XPB and p8 mutation L21P (TTD)) localize to the collar structure (Fig. 7e) formed by dynamic communities D (NTE, p52), O (DRD) and K (p8, p52). These mutations would not impact ATP hydrolysis, but by affecting the dynamic coordination of the XPB ATPase domains could result in partly deficient dsDNA translocation activity.

## Discussion

TFIIH mechanisms critically depend on XPB and XPD – two ATPases with opposite polarities. To unravel the precise functional roles of XPB and XPD in the TFIIH molecular machinery, we built suitably complete structural models of TFIIH in transcription and NER-competent states. We then used extensive MD simulations (ModelArchive accession code: [https://www.modelarchive.org/doi/10.5452/ma-2chon]; Supplementary Data 3) and novel graph-theoretical methods to analyze the functional dynamics of these assemblies.

While XPB and XPD each have independent helicase activities on DNA, they serve different functions in TFIIH and are regulated differently depending on functional context (e.g., transcription versus NER). During transcription initiation, XPB uses its translocase activity to rotate and push the DNA duplex upstream of Pol II toward the RNA polymerase cleft. This induces severe kinking and unwinding of dsDNA within the cleft, which eventually results in the formation of the nascent transcription bubble. In this context, XPD plays a purely structural role in ensuring PIC structural integrity and stable XPB association to the rest of the initiation machinery. Consequently, XPD’s intrinsic ATPase activity and internal dynamics are suppressed by the insertion of a p62 segment and an autoinhibitory loop into the XPD DNA-binding groove.

**Fig. 8:**
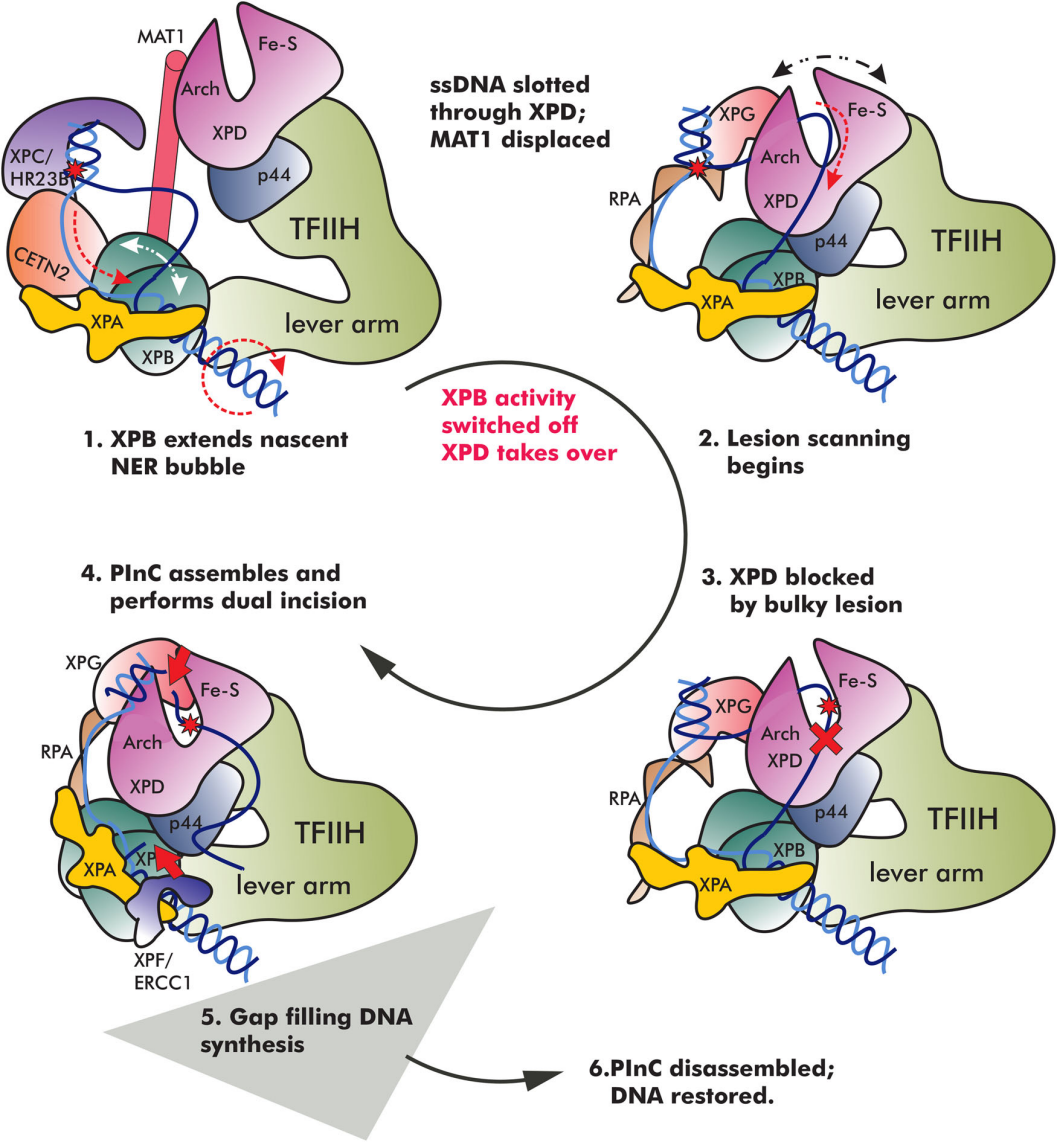
Conformational switching mechanism enabling sequential coordination of XPB and XPD activities in NER. The schematic represents key steps in the NER pathway—XPC lesion recognition, NER bubble extension, XPD-mediated lesion scanning, PInC assembly and DNA incision by XPG and XPF/ERCC1, gap filling synthesis, and DNA restoration. Protein participants in NER are shown as cartoons, color-coded, and labeled. The DNA lesion position is shown as a red star. Red dashed arrows indicate the direction in which ssDNA is displaced during the different stages of NER and the rotation of the DNA duplex by XPB. White dotted arrow indicates the displacement of the XPB ATPase domains. Black dotted arrow indicates the opening/closing of the XPD Arch and Fe–S domains during ssDNA translocation. A red cross indicates the blocking of a bulky lesion inside XPD and damage verification. Two red arrows indicate the incision points on DNA by XPF/ERCC1 and XPG.

By contrast, during GG-NER XPB extends the nascent bubble created by XPC^13^ while XPD uses its ssDNA translocase activity for lesion scanning. In this context, the ATPase activities of both XPB and XPD are indispensable. A long-standing notion in the NER field has been that XPB and XPD cooperate in opening the DNA bubble. Here we show that the action of XPB and XPD is sequential rather than cooperative. In our computationally informed mechanism (Fig. 8), XPB acts first to unwind DNA downstream of the lesion site, which is recognized by XPC/HR23B/CETN2 and held at the 3′ edge of the expanding DNA bubble. Recruitment of XPA, stimulates XPB unwinding by clamping dsDNA and preventing its dissociation from XPB. XPB functions as a translocase, which simultaneously rotates the downstream duplex and pushes it toward XPA. An XPA β-hairpin splits the two DNA strands. The high affinity of XPD for ssDNA results in slotting of the lesion-carrying strand into the XPD groove once the bubble reaches a critical size. This triggers a series of conformational switches involving MAT1 displacement by the N-terminus of XPA, repositioning of p62 and the collapse of the spacing between XPB and XPD. Indeed, it has been previously proposed that MAT1 could serve as XPB–XPD spacer and MAT1 removal could allow XPD to approach DNA^13^. The binding of XPD/p44 to both lobes of XPB blocks dsDNA translocase activity. Afterward, XPD starts to reel in ssDNA in the 3′ to 5′ direction, unwinding the upstream DNA duplex. When the lesion reaches the constriction between the XPD Arch and Fe–S domains unwinding stops, allowing the PInC complex to assemble and carry out dual incision of the lesioned strand^76^. Crucially, the XPB and XPD activities of TFIIH are sequentially coordinated and mutually exclusive during NER to achieve precise DNA incision. Limiting XPB unwinding to the early stages of NER prevents accumulation of excess ssDNA between the two translocases. Thus, our model explains the observed narrow size distribution of excision products^49,77^.

Our findings provide a long sought in-depth insight on the etiology of TFIIH-associated severe genetic disorders-xeroderma pigmentosum, trichothiodystrophy and xeroderma pigmentosum/Cockayne syndrome^55–58^. Strikingly, we find that disease mutations map onto key dynamic community interfaces. Importantly, to differentiate mutations by phenotype it is necessary to consider TFIIH dynamics in multiple pathways and functional states. Our new network models incorporate data from multiple MD ensembles and account for TFIIH’s dramatic structural reorganization. Considering both the PIC and GG-NER functional states, practically all XP, XP/CS, TTD and XP/TTD mutants localize to community interfaces. They are positioned to impair functionally relevant dynamic motions necessary for transcription (TTD), nucleotide excision repair (XP, XP/TTD) or both (XP/CS). The idea of augmenting our network models with data from multiple ensembles sets the stage for future studies that would address the multitude of TFIIH conformational states. Specifically, we expect that binding and exchange of repair factors would drive functionally important TFIIH conformational changes at different stages of the GG-NER or TC-NER pathways. These repair factors include XPC, XPA, XPG, RPA and ERCC1/XPF for GG-NER^1,50,76^ and CSB, CSA, RBX1, CUL4A, DDB1 in TC-NER^11,78^. Analysis of these additional states would further contribute to our understanding of the link between mutations and disease phenotypes.

Collectively, our results inform the intricate and coordinated molecular choreography of TFIIH and the regulatory mechanisms underpinning its diverse functions in gene expression and genome maintenance. Our methods and models provide a framework for future experiments to tackle the complex interplay of TFIIH structure, dynamics, and disease phenotypes.

## Methods

### Model building

To model the NER-TFIIH complex, we used the existing TFIIH–XPA–DNA cryo-EM density (EMDB accession code: EMD-4970 [https://www.ebi.ac.uk/emdb/EMD-4970])^32^. The p62 subunit of TFIIH and the XPA N-terminal extension (residues 1–103) and C-terminal extension (residues 238–273) had no known structural homologs and were built de novo. The Genesilico metaserver^79^ was used for consensus secondary structure prediction, which allowed us to determine the sequence register in the EM density. To model p62 (residues 107–548), we started with the subunit conformation from the PIC (PDB accession code: 6O9L [https://www.rcsb.org/structure/6O9L]), which was then docked and repositioned into the EMD-4970 density using COOT^80^. The missing loop regions of XPB, XPD, p44, p34, and p52 were also modeled into the EM density. The TFIIH–XPA–DNA structure was then refined in a 10-ns simulation using the molecular dynamics flexible fitting (MDFF) method^81–83^.The MDFF biasing potential was applied with a scaling factor ξ of 0.3.

### Molecular dynamics

Molecular dynamics simulations of the TFIIH complexes in distinct functional states were carried out on the Summit machine at the Oak Ridge Leadership Computing Facility. All systems were set up with the TLeap module of AMBER^84^ and solvated with TIP3P water molecules^85^. Na^+^ counterions were introduced to neutralize the overall charge on each system. Additional Na^+^ and Cl^−^ ions were added to ensure 150 mM salt concentration and mimic physiological conditions. Energy minimization was performed with the NAMD code for 5000 steps with positional constraints imposed on the backbone atoms of all protein and nucleic acid chains. Afterward, an NVT simulation run was used to gradually bring the temperature of each system from 0 to 300 K over a period of 100 ps. During this time, we imposed positional restraints (*k* = 10 kcal mol^−1^ Å^−2^) on all heavy atoms of the protein complex. We continued the equilibration of the models for another 5 ns in the NPT ensemble while gradually releasing the positional restraints. Production runs were performed in the NPT ensemble (1 atm, 300 K) for 1µs for the apo-TFIIH, holo-PIC and NER-TFIIH complexes. In the simulations, the particle mesh Ewald (PME) method was used to evaluate long-range electrostatic interactions. The r-RESPA multiple-time-step method^86^ was employed with a 2-fs timestep for bonded interactions, a 2-fs timestep for short-range non-bonded interactions and 4-fs timestep for long-range electrostatic interactions. A short-range non-bonded interaction cutoff of 10 Å and a switching function at 8.5 Å were used for the simulations. All covalent bonds to hydrogen atoms were constrained using the SHAKE algorithm. The simulations were carried out with the NAMD 2.14 code^87^ and the AMBER forcefields: Parm14SB^88^ and OL15^89^. All figures were generated using UCSF Chimera^90^.

### Difference contacts network analysis and principal component analysis

Contact maps for the TFIIH complexes were generated from the MD ensemble data with the MDTraj package^91^. The consensus network was obtained from the contact maps of apo-TFIIH, NER-TFIIH and PIC-TFIIH. To find the network of communities, the consensus network was continually subdivided using a non-weighted Girvan-Newman algorithm^92^, using the Python package NetworkX, until the difference in modularity between subsequent partitions was <0.001. The final partition resulted in 17 distinct communities with a modularity of 0.91. The change in the contact probability between the consensus communities was then computed for the PIC → apo and the apo → NER conformational transitions. This was done by subtracting the respective contact maps for the two end states to obtain difference networks. The overall probability change, Δ*P*, between communities was determined by summing the weights of the edges between pairs of communities. As contact probability changes for pairs of individual residues can be either positive or negative, the same is true for the overall change Δ*P*. Positive Δ*P* signifies increase in interactions between communities during the conformational transition. Conversely, negative ΔP signifies decrease in interactions. PCA was performed on the MD ensemble trajectory data for the NER-TFIIH. PCA was performed using the CPPTRAJ module in AmberTools16^93^.

### Path optimization with the partial nudged elastic band method

To explore the DNA translocation mechanisms of XPB and XPD, we employed chain-of-replicas path optimization methods. We used the partial nudged elastic band method (PNEB)^64^ to compute minimum free energy paths for the XPB and XPD conformational transitions between nucleotide free, ATP- and ADP-bound states along the respective ATP hydrolysis cycles. Each path was comprised of 70 replicas representing the intermediates in the ATP hydrolysis cycle. For human XPB, PDB structures were available for all nucleotide states, which allowed us to initiate PNEB modeling directly. Specifically, we modeled ATP-bound XPB from the post-translocated state of XPB in TFIIH (determined with the ATP analog ADP-BeF3) (PDB ID: 7NVV [https://www.rcsb.org/structure/7NVV]^94^). For human XPD, no ATP-bound structure was available. Therefore, we modeled ATP-bound XPD based on the *Escherichia coli* dinG structure (PDB ID: 6FWS [https://www.rcsb.org/structure/6FWS]^63^). Apo XPB and XPD were taken from TFIIH pre-translocation state (PDB ID: 7NVW [https://www.rcsb.org/structure/7NVW]) and TFIIH–XPA-DNA complex (PDB ID: 6RO4 [https://www.rcsb.org/structure/6RO4]), respectively.

For each system, XPB and XPD, we computed independent PNEB paths for three ATP hydrolysis cycles representing three consecutive steps in the translocation mechanisms. The initial and end states of each cycle corresponded to the equilibrated apo state of XPB and XPD, respectively. The ATP- and ADP-bound states were introduced as intermediates in each cycle. All heavy atoms of the complexes were included in the path optimization. The systems were heated from 0 to 300 K using the Langevin dynamics thermostat (collision frequency of 1000 ps^−1^) while applying PNEB spring forces between neighboring images of 10 kcal mol^−1^ Å^−2^. PNEB production runs were carried out at 300 K for ~10 ns. Convergence of the band was monitored by the change in protein backbone RMSD values of the replicas during the simulations.

### Traditional (Covariance-based) community network analysis

Covariance-based community network analysis was performed on the TFIIH-NER trajectory. The MDTraj package^90^ was used to obtain the contact map for the ensemble. In this network analysis method, the nodes in the network represent the Cα and P atoms of the protein or DNA residues. The edges of the network represent the contacts between the residues. Two non-adjacent residues are considered to be in contact if they are within 4.5 Å for 75% or more of the trajectory. The edges have weights (*w*_*i,j*_) given by: ${w}_{i,j}=-{{{{{\rm{ln}}}}}}(|{c}_{i,j}|)$, where *c*_*i,j*_ is the correlation coefficient for the residue pair. The Girvan–Newman algorithm^91^ was used to partition the weighted protein network graph by iteratively removing loosely connected edges. This resulted in partitioning the TFIIH-NER ensemble into 16 dynamic communities with a modularity score of 0.918.

### Rosetta protein stability analysis

TFIIH disease mutations were assessed for their effect on protein stability using the Rosetta Cartesian ddG protocol^75^. The wild type (WT) structures of the NER complex were relaxed in cartesian space using the Rosetta FastRelax protocol. Mutations are then introduced and the FastRelax protocol used to repack the side chains within 6 Å of the mutation site. The backbone within 3 residues of the mutation site is also allowed to move. The ddG value is determined by the Rosetta score difference between the relaxed mutant protein and the relaxed WT protein.

### Reporting summary

Further information on research design is available in the Nature Portfolio Reporting Summary linked to this article.

## Supplementary information

Supplementary Information

Description of Additional Supplementary Files

Supplementary Data 1

Supplementary Data 2

Supplementary Data 3

Supplementary Movie 1

Supplementary Movie 2

Supplementary Movie 3

Supplementary Movie 4

Supplementary Movie 5

Reporting Summary

## Data Availability

The data that support the findings of this study are available from the corresponding authors upon reasonable request. The model of TFIIH-NER complex has been deposited in the ModelArchive database with DOI accession code: 10.5452/ma-2chon. The list and functional annotation of TFIIH disease mutations generated in this study are provided as Supplementary Data 1 file. The Rosetta ddG scores generated in this study are provided as Supplementary Data 2 file. The final configuration of the TFIIH-NER molecular dynamics trajectory is provided as a plain text file TFIIH-NER-complex-final-MD-configuration_PDB.txt in PDB format as Supplementary Data 3 file. Accession codes of all the publicly available datasets used in the study: PDB accession codes 6O9L, 7NVV, 6FWS, and 7NVW and EMDB accession code EMD-4970.
