# Supplementary material for: Dynamic conformational switching underlies TFIIH function in transcription and DNA repair and impacts genetic diseases
Source: Nat Commun. 2023 May 13;14:2758. doi: 10.1038/s41467-023-38416-6 (PMC10183003; doi:10.1038/s41467-023-38416-6)
Supplement: Supplementary file 1 — Supplementary Information [file 41467_2023_38416_MOESM1_ESM.pdf]

SUPPLEMENTARY FIGURES

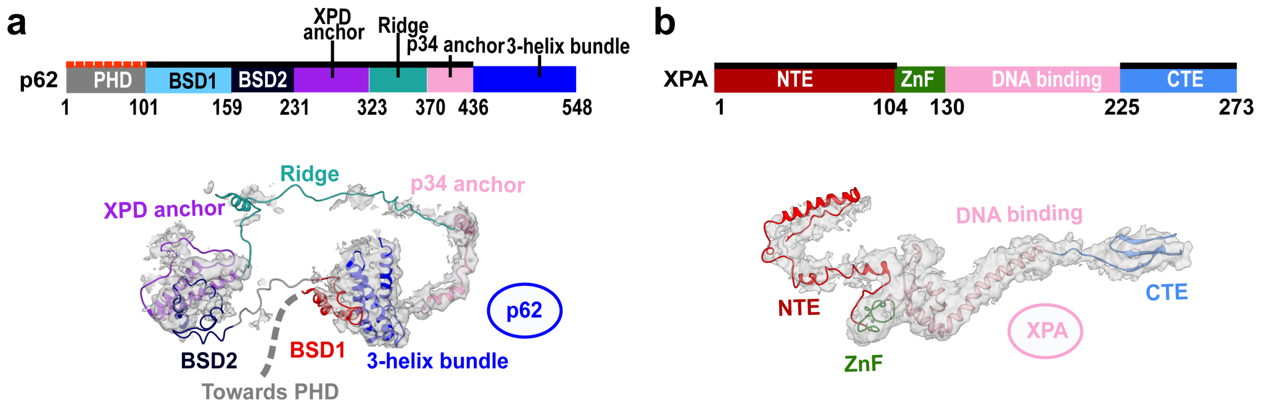

Supplementary Figure 1. Regions of the NER-TFIIH complex modelled *de novo*. Newly modeled regions in **a,** the p62 subunit and **b,** XPA. The schematics at the top of each panel shows the domains and structural motifs of p62 and XPA mapped onto the respective sequences. Structures are depicted in cartoon representation and colored by domain. Solid black lines in the schematics demark the newly modeled regions.

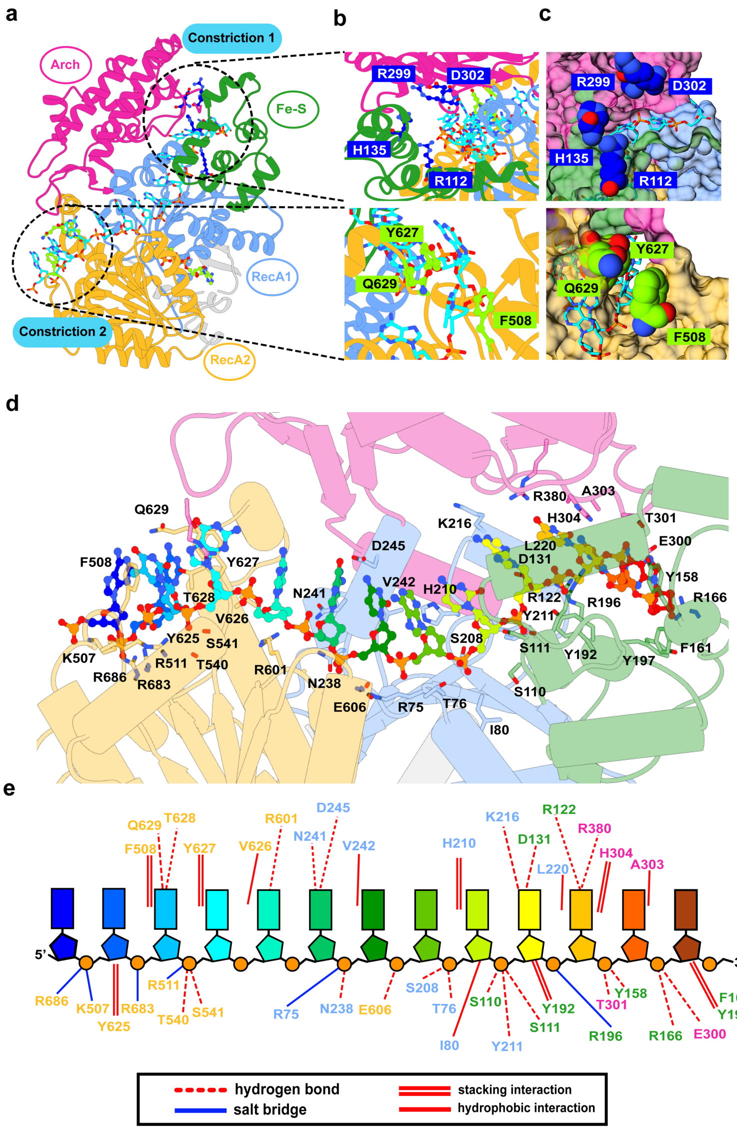

Supplementary Figure 2. Protein-ssDNA contacts from MD reveal the mode of XPD-DNA association. a, Structure of the XPD-ssDNA complex in the apo-state. The RecA1, RecA2, Fe-S, and Arch domains are shown in light blue, dark green, orange, and dark red, respectively. The two constrictions along the DNA path in XPD are indicated by showing the key residues involved (green for Constriction 1; and blue for Constriction 2). **b,** Zoomed view of Constriction 1 (top) and Constriction 2 (bottom). **c,** Constriction 1 (top) and Constriction 2 (bottom) in surface representation (same view as in **b**). **d,** XPD–DNA contacts along the XPD DNA-binding groove. XPD is shown in cartoon representation and colored by domains. Residues participating in ssDNA binding are shown in all atom representation. **e,** Schematic detailing key contacts between XPD and DNA classified by interaction type (hydrogen bond, salt bridge, hydrophobic or stacking). Residue labels are colored by domain. DNA phosphate groups are shown as black spheres.

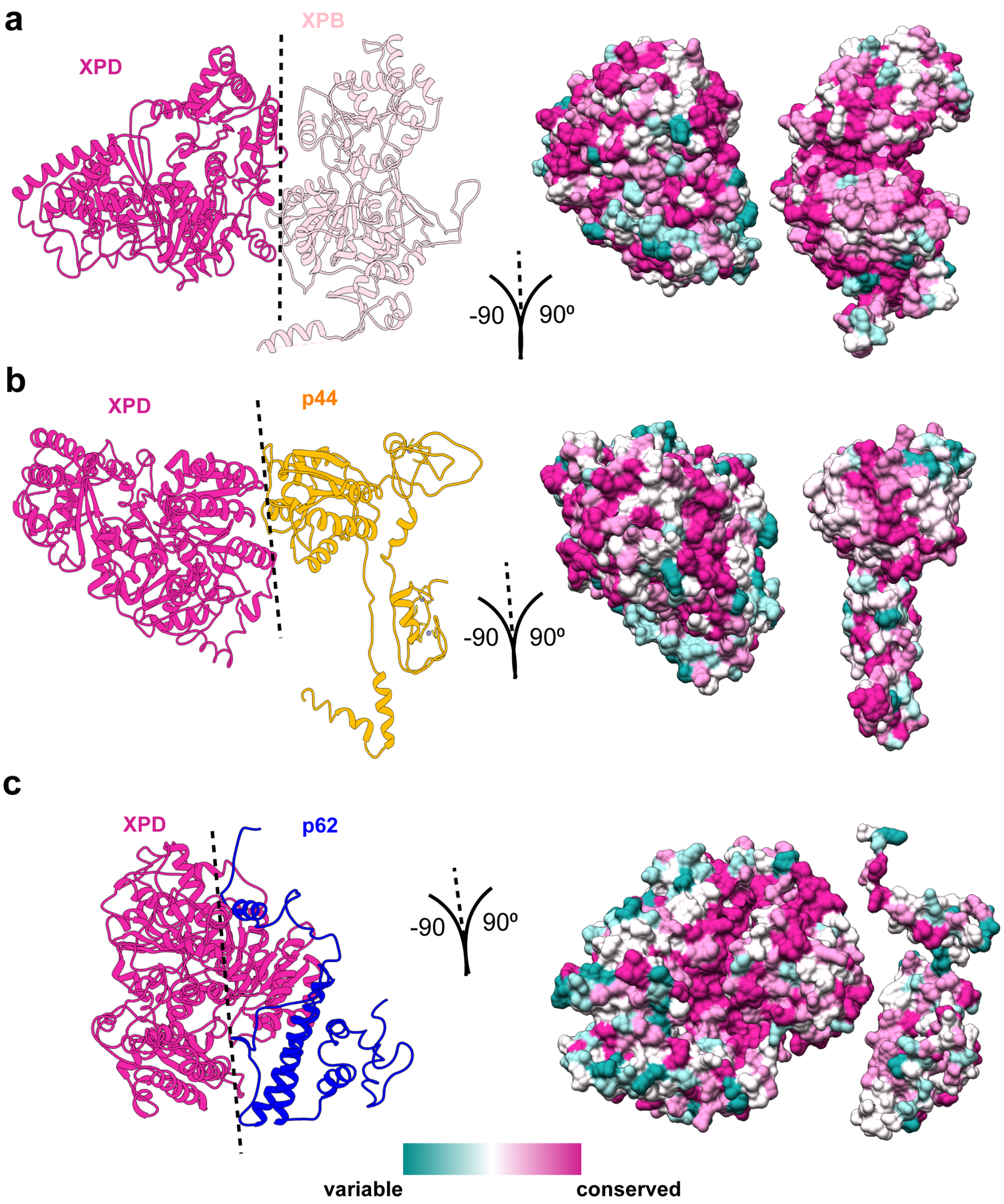

Supplementary Figure 3. Conservation maps of the XPD/XPB, XPD/p44 and XPD/p62 interfaces in the TFIIH-NER complex. Structures and corresponding conservation maps of the **a,** XPD/XPB interface. **b,** XPD/p44 interface and **c,** XPD/p62 XPD anchor interface. Conservation is mapped onto the molecular surface of the proteins. The interfaces are opened and rotated for easy viewing. Conservation maps are based on alignment of the following sequences: human XPD, p44, p62 and corresponding homologs respectively of *Xenopus laevis*, *Mus musculus*, *Podarcis muralis*, *Drosophila melanogaster* and, *Saccharomyces cerevisiae.*

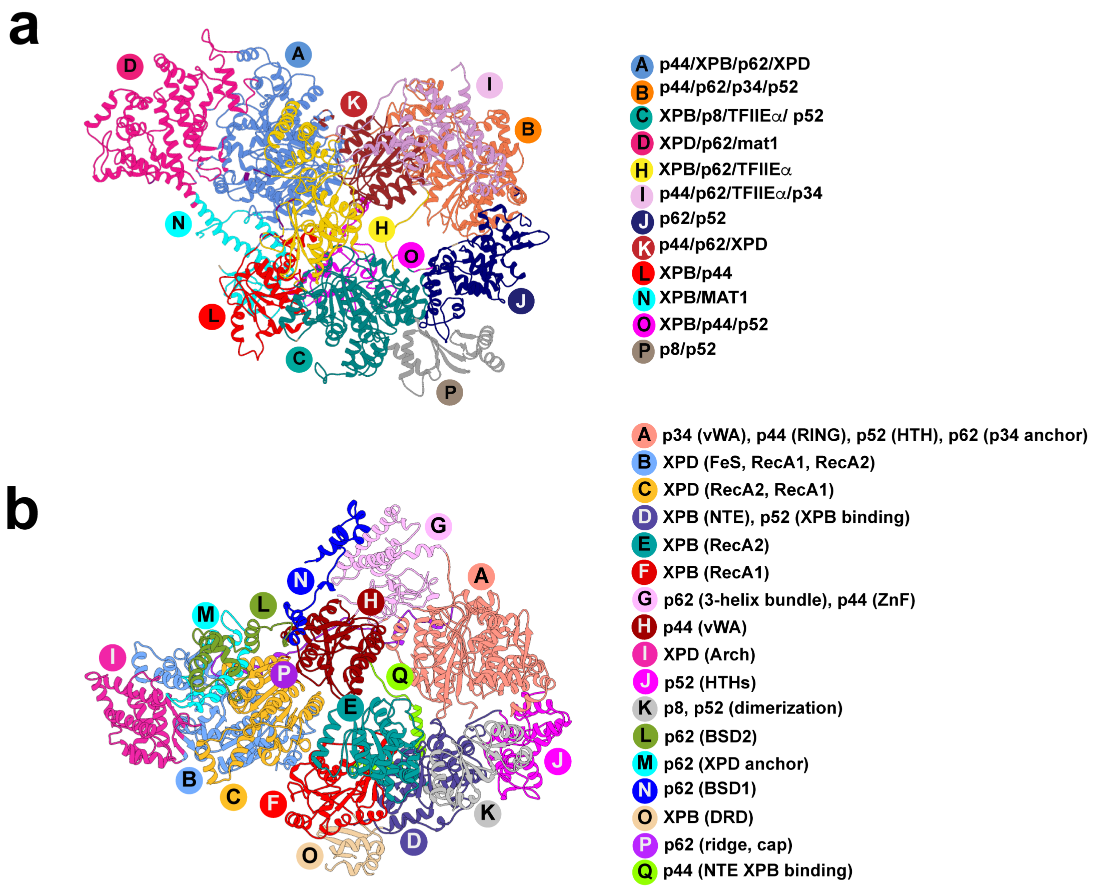

Supplementary Figure 4. Comparison of the TFIIH community networks for the holo-PIC complex and NER-TFIIH complex from conventional network analysis. a, Holo-PIC communities identified from network analysis using dynamic cross correlation data from MD; **b,** NER-TFIIH communities identified from network analysis using dynamic cross correlation data from MD. Communities are mapped on the structures of holo-PIC and NER-TFIIH respectively, and are color-coded and labeled.

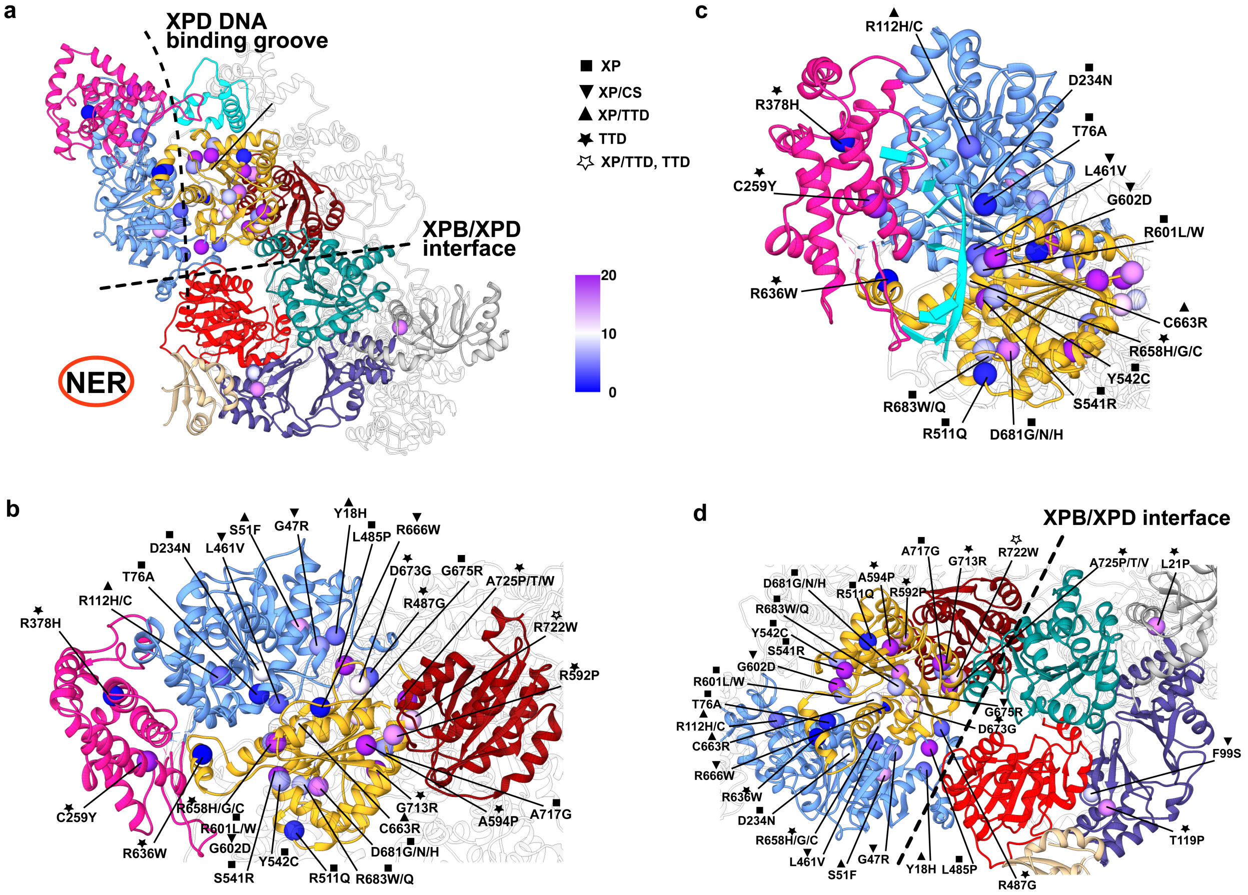

Supplementary Figure 5. TTD disease mutations affect TFIIH stability. **a**, TTD, XP, XP/CS and XP/TTD point mutations mapped onto XPD, XPB, and p8 subunits and colored by Rosetta ddG scores; **b**, Zoomed view of mutations within XPD; **c**, Zoomed view of mutations lining the path of ssDNA in XPD; **d**, Zoomed view of mutations at the XPB-XPD interface.
