# Supplementary material for: Dynamic conformational switching underlies TFIIH function in transcription and DNA repair and impacts genetic diseases
Source: Nat Commun. 2023 May 13;14:2758. doi: 10.1038/s41467-023-38416-6 (PMC10183003; doi:10.1038/s41467-023-38416-6)
Supplement: Supplementary file 2 — Description of Additional Supplementary Files [file 41467_2023_38416_MOESM2_ESM.pdf]

Description of Additional Supplementary Files

File Name: Supplementary Data 1
Description: Human Disease Mutations in TFIIH-NER – Consensus Community-based Analysis.

File Name: Supplementary Data 2
Description: Computed Rosetta ddG values of TFIIH disease mutations for the PIC and TFIIH-NER complex (Source Data File for Supplementary Figure 5).

File Name: Supplementary Data 3
Description: Final configuration from the molecular dynamics trajectory of the TFIIH-NER complex supplied as a plain text file in PDB format.

File Name: Supplementary Movie 1
Description: **Structural reorganization of the TFIIH assembly from holo-PIC to apo- TFIIH and NERTFIIH.** TFIIH is colored by domains: XPD is shown in magenta, XPB in pink, p44 in dark red, p62 in blue; p62's XPD anchor in green.

File Name: Supplementary Movie 2
Description: **Mechanism of XPB translocation on duplex DNA.** We visualize the translocation process in its entirety as represented by the minimum free energy path computed with the partial nudged elastic band method. XPB is colored by domains: RecA1 is shown in red, RecA2 in dark cyan, NTE in purple, DRD in tan, dsDNA in cyan. The ATP and ADP are colored in cyan and purple, respectively.

File Name: Supplementary Movie 3
Description: **Mechanism of XPD translocation on singlestranded DNA**. We visualize the translocation process in its entirety as represented by the minimum free energy path computed with the partial nudged elastic band method. XPD is colored by domains: RecA1 is shown in light blue, RecA2 in gold, Fe-S in green, Arch in red. The ATP and ADP are colored in cyan and purple, respectively. ssDNA is shown explicitly and colored by nucleotide in the 5' to 3' direction (from blue to red). Constriction 1 and Constriction 2 are shown in ball and stick representation and colored in light green and blue, respectively.

File Name: Supplementary Movie 4
Description: **Simulations reveal that XPD and p8 are the only constituents of NER- TFIIH assembly participating in large-scale dynamic motions.** We visualize the first mode (PC1) from principal component analysis of the NER-TFIIH complex. Select dynamic communities of the NER-TFIIH complex are colored as denoted in the video inset.

File Name: Supplementary Movie 5
Description: **Mapping of disease mutations onto the TFIIH consensus community structure and shown in the context of the structural reorganization of TFIIH from holo-PIC to apo-TFIIH and NER-TFIIH.** Select TFIIH communities are colored as denoted in the video inset. Human disease mutations are represented as spheres and colored by phenotype.
