# Supplementary material for: Dynamic conformational switching underlies TFIIH function in transcription and DNA repair and impacts genetic diseases
Source: Nat Commun. 2023 May 13;14:2758. doi: 10.1038/s41467-023-38416-6 (PMC10183003; doi:10.1038/s41467-023-38416-6)
Supplement: Supplementary file 3 — Supplementary Data 1 [file 41467_2023_38416_MOESM3_ESM.pdf]

Table S1: Human Disease Mutations in TFIIH-NER – Consensus Community-based Analysis

Protein	Mutation	Disease*	Consensus Community Interface	Interactions Affected
P8	L21P	TTD	K/E	DOI: 10.1038/ng1387. L21 is a solvent accessible hydrophobic located in the middle of a helix that packs against XPB. In the NER complex, it also connects to XPA. L21P is active for transcription but NER defective, DOI: 10.1038/ng1387. Mutation to P would disrupt the helix and remove L21 aliphatic packing against I34 and K17, likely disrupting TFIIH structural integrity between p8 and XPB, explaining the TTD phenotype.
XPB	F99S	XP/CS	D/F<br>D/Q	PMID:8304337. F99 is located centrally within a helix that is braced against the p44 N-terminal helix in all three complexes. The side chain falls into a hydrophobic pocket. Both the p44 helix and XPB F99 helix rotate in PIC compared to the apo or NER complexes, with F99 directly contacting Y15. Mutation to S will replace a bulky hydrophobic with a much smaller polar residue, which is likely to destabilize the region.
XPB	T119P	TTD	D/F<br>D/O	Weeda, 1997 (PMID: 9012405). T119 is at the end of a beta strand and N-caps a short helix. This XPB mini-domain interacts with p52 and p44. In addition to changing the bond angles in the tight turn from strand to helix, the mutation from T to P would remove the cap and likely destabilize the short helix. This helix is close to the XPB/p44 NTE interface and therefore could disrupt the interface.
XPD	Y18H	XP/TTD	B/F	DOI:10.4161/rdis.24932.Y18 is at the end of a helix and in the apo complex H-bonds to the G45 backbone (two residues from XP/CS mutation at position 47). In the apo and PIC complexes, Y18 is no longer interacting with the p62 ridge (comm P), but is at a new interface between the XPD RecA1 and XPB RecA1 domains (comm F). Mutation to His in apo and PIC will likely disrupt p62 interaction causing TTD, while His mutation in the NER complex likely disrupts the hydrophobic core with LEU738 and LEU485 and the helicase activity.
XPD	G47R	XP/CS	B/C	DOI: 10.1111/j.0022-202X.2005.23745.x, 10.1093/hmg/3.10.1783, PMID: 25716912.<br>G47 is in a helicase motif and is located near Y18 (site for mixed XP/TTD). Similarly to Y18, in the NER complex, it has also lost the interface with the p62 ridge (comm P). G47 lies at the interface between the XPD RecA1 and RecA2 domains for all three complexes. In the apo and PIC complex, the mutation would disrupt the p62 interaction. In the NER complex, mutation

				would disrupt ATP binding. Moreover, mutation to R will likely rigidify the end of the helix.
XPD	S51F	XP/TTD	B/C	DOI: 10.1002/humu.20768. S51 packs in tight pocket with H-bonds to backbone carbonyl (res 48 i-3 – next to G47 XP/CS mutant side) and helps terminate the helix. See DOI: 10.1016/S0006-3495(00)76514-3. In the apo and PIC complexes, S51 is 2-3 residues from the interface to p62. However, in NER, this interface with the p62 ridge/cap is lost (comm P). S51 neighbors the ATPase motifs, and mutation to bulky F is likely to disrupt ATPase activity by interfering with the helix packing, explaining XP. In the apo and PIC complexes, mutation will disrupt p62 interaction, explaining TTD.
XPD	T76A	XP	B/C<br>B/P	DOI: 10.1101/gad.859501. T76 is a beta branched helix breaker that helps end the helix. In the transition to NER, T76 is at the RecA1/RecA2 interface and has new ssDNA interactions with its amino polar region facing the phosphodiester. This rearrangement also results in T76 switching interactions from the p62 XPD anchor (comm M) to the p62 cap/ridge (comm P). Alanine mutation cuts helicase activity (DOI: 10.1016/j.cell.2008.04.030) by decreasing the rigidity of that region. Mutation to A would also likely disrupt hydrogen bonding with the DNA backbone, thereby explaining the XP phenotype.
XPD	R112H/C	XP/TTD	B/P	DOI: 10.1101/gad.859501, 10.1002/humu.20768, 10.1093/hmg/10.22.2539. In the NER complex, R112 is in the Fe-S domain with direct contacts to the DNA phosphodiester and may protect the Fe-S cluster, explaining reduced helicase activity (DOI: 10.1016/j.cell.2008.04.030) and XP phenotype when mutated. In the apo and PIC, R112 is 1-2 residue layers away from p62, therefore mutation may impact assembly and explains TTD phenotype.
XPD	D234N	XP	B/C	DOI: 10.1101/gad.859501. D234 (in the Walker B motif) binds Mg for ATP hydrolysis. N mutation would disrupt Mg coordination and reduce helicase activity (DOI: 10.1016/j.cell.2008.04.030) for repair.
XPD	C259Y	TTD	I	DOI: 10.1101/gad.859501. C259 is deep within a helix in a tightly packed hydrophobic region. In the NER complex, this helix is packed against XPA and p62. In the apo and PIC complexes, this residue is 1-2 residue layers from p62 and 2-3 layers from MAT1. Mutation to the bulky Y residue would destabilize the helical interface by creating steric clashes with the surrounding hydrophobic residues, explaining the TTD phenotype.
XPD	R378H	TTD	I	DOI: 10.1002/humu.20768. R378 is involved in helix packing with V289, L382, and A326 with amides H-bonding to S381. In the apo and PIC complexes, R378 makes a buried salt bridge with E284 as well as

				aliphatic packing against F329 and F374. R378 is one residue layer away from MAT1 in the PIC and apo and from XPA in NER. Thus, the disruption from the loss of hydrophobic interactions and a buried negative charge will likely destabilize the complex.
XPD	L461V: (del716-730) A717G	XP/CS	B/C	DOI: 10.1038/jhg.2015.18, 10.1111/j.0022-202X.2005.23745.x, 10.1016/j.ccr.2006.05.027, 10.1073/pnas.94.16.8658. Explanation of the L461V as a single site mutation is complicated. It is found on the same allele with a second mutation, c.2150 C > G that generates a cryptic splice donor site and is expected to cause abnormal splicing resulting in a double mutant L461V; V716_R730del. The deletion region V716 to R730 forms the interface with p44 and XPB, suggesting that TFIIH would not be able to properly assembly and consistent with the lack of stability of mutant V716-R730del in cells. This double mutant, L461V; V716_R730del, is likely null. Normal splicing can also occur (based on RT-PCR), resulting in a distinct double mutant L461V: A717G. In terms of structure, L461 and A717 are 30 Å away from each other, on opposite sides of the RECA1 domain and their effects appear to be cumulative, since individual mutations have been shown to retain function against UV while the double mutant is defective. Evidence that L461V: A717G can partially contribute function is that patients have milder XP-D/CS as a compound heterozygote with G47R or R666W, when compared to other G47R or R666W compound heterozygotes. L461 is in a helicase motif that packs against V39 and I456. V mutation would impact the helicase, by making the region more rigid due to branched beta carbon, and may also lose some side chain interactions due to having a smaller side chain. In addition to XP/CS, the L461V plus del 716-730 (or double mutant L461V: A717G) mutation is associated with XP (XP84BE, second allele: R683W), with TTD (TTD412BE, second allele: A725P), and with XP/TTD (XPTTD526BE, second allele: R196P).
XPD	L485P	XP	B/C<br>B/F	DOI: 10.1093/hmg/10.22.2539. L485 is in a loop, packing with F728 near the helicase domain interface. In the NER complex, L485 lies at the interface of the XPD RecA1/RecA2 domains as well as the newly formed XPD RecA1/XPB RecA1 interface. It is likely involved in rigidifying the interface to inhibit XPB helicase activity. Mutation to P would destabilize the backbone and possibly affect the interaction of the two XPD helicase domains and the XPD/XPB interaction.

XPD	R487G	TTD	C/B<br>C/F<br>C/H	DOI: 10.1101/gad.859501. R487 is in a loop near the p44 interface and provides aliphatic stacking with F728 and backbone H-bonding with M724 and Q726. It also C-caps the helix in the p44 interface. In the apo and PIC complexes, it is close to D673 (TTD mutation site) and p62. Mutation to G removes side chain interactions and main chain stability, which would disrupt the dynamic community interface.
XPD	R511Q	XP	C/M<br>C/L	DOI: 10.1101/gad.859501. R511 is in the DNA binding groove. Mutation to Q would reduce DNA binding and helicase activity, explaining the XP phenotype.
XPD	S541R	XP	C/M	DOI: 10.1101/gad.859501. S541 is next to Y542C (XP) which is in a helicase motif. In the NER complex, it N-caps a helix flanking the DNA binding groove. It is noteworthy that the amino-polarized helical end is facing the negatively charged phosphodiester backbone, so S541 could be playing an important role in positioning the end of the helix at the edge of the DNA binding groove and orienting the helix dipole to attract the ssDNA backbone. Mutation to R likely reduces helix cap stability and impacts DNA binding near the p62 XPD anchor interface.
XPD	Y542C	XP	C/M	DOI: 10.1101/gad.859501. Y542 is in a helicase motif that packs against aliphatic chains of Q572 and V599 to orientate a helix N-terminus. The Y side chain would stack against the DNA backbone in the NER complex. It is near S541 (XP) and p62 XPD anchor binding interfaces. Mutation to C creates a destabilizing void under the DNA-binding groove.
XPD	R592P	TTD	C/H<br>C/M	DOI: 10.1101/gad.859501. In all three structures, R592 is in a helicase motif that is a C-cap for a helix and in the interface with p44. In the PIC complex, R592 interacts with K161 of p62. In the NER complex, R592 makes a salt bridge to E142 of p44, but lacks the p62 interface. In the apo complex, R592 makes contacts to both p44 and p62. Mutation to P removes these interactions and likely disrupts this region by backbone distortion, explaining the TTD phenotype.
XPD	A594P	TTD	C/H	DOI: 10.1101/gad.859501. A594 is at the end of a beta strand and is in a helicase motif at the junction of the XPD and p44 interface. It is packed with L566, F568, Y582, and C588 in a hydrophobic pocket. A594 packs near p62 in the PIC and apo structures, but does not interact with p62 in the NER structure. Mutation to P would disrupt packing and backbone conformation at the XPD/p44 interface, explaining the TTD phenotype.
XPD	R601L/W	XP	C/B	DOI: 10.1101/gad.859501. R601 is in a helicase motif loop near two XP mutations (541 and 542), the DNA binding groove, and has interactions with a helix near the ATP binding pocket in the NER complex. R601 also

				loses the interface with the p62 XPD anchor domain that was present in the PIC. The R aliphatic chain packs against H659 and V626 and it forms a salt bridge to D655 near the interface between helical domains and the Walker B motif. L or W mutation disrupts packing will lead to an unfulfilled negative charge on D655 in the DNA binding groove, likely disrupting DNA binding and explaining the XP phenotype (see DOI: 10.1016/j.cell.2008.04.030).
XPD	G602D	XP/CS	C/B	DOI: 10.1101/gad.859501. G602 is in a helicase motif loop at end of a helical turn adjacent to the DNA binding groove. Similar to R601, the interface with p62 XPD anchor is lost in the NER complex and replaced with the DNA interface. Mutation to D would reduce structural flexibility and create a negative repulsion with both the DNA backbone and with E570 in a neighboring loop.
XPD	R636W	TTD	C/B<br>C/I	Shin, 2013, PMID: 23884229. R636 is in the middle of a helix at the interface of XPD RecA2/RecA1. Its aliphatic chain packs against L640 and M438. R636 forms a salt bridge with D240 near XP mutant R658. Mutation to W would disrupt this packing and salt bridge.
XPD	R658H/G/C	TTD	C/B	DOI: 10.1101/gad.859501. Takayama, 1996, 8571952. R658 is in a helicase motif and helix at the interface of the XPD helicase domains. It makes a salt bridge to D240 and D655 next to the XPD Walker B motif. Its side chain packs against F654 and F651 adjacent to the XPB interface in the apo structure and XPA in the NER structure. H, G, or C mutation will not support the network of salt bridges and prevent the packing to the two F side chains and weaken the structural integrity of the XPD/XPB interface in .
XPD	C663R	XP/TTD	C/B	DOI: 10.1101/gad.859501, 10.1002/humu.20768. C663 is in a helicase motif and helix on the interface between the XPD RecA2 and RecA1 domains close to the ATP binding pocket, with its side chain in a hydrophobic pocket and packing with F538 and A600. R mutation likely distorts the helix and interaction between communities.
XPD	R666W	XP/CS	C/B	DOI: 10.1101/gad.859501, 10.1038/jhg.2015.18, 10.1111/j.0022-202X.2005.23745.x, 10.1016/j.ccr.2006.05.027. R666 is in a helicase motif and would bind the gamma phosphate of ATP. It would C-cap the end of a helix, stabilizing the XPD structure. R666 also extends into a pocket where it forms a salt bridge to E235 in Walker B motif. W mutation removes ATPase and helicase activity (see DOI: 10.1016/j.cell.2008.04.030) and disrupts interactions while increasing local rigidity by filling the

				pocket.
XPD	D673G	TTD	C/B<br>C/H	DOI: 10.1101/gad.859501. D673 is on a loop at the end of a beta strand with likely interactions to R669 and H612. Mutation to G could result in a loss of interaction of H612, which could affect the interface with p44. In the PIC complex, R669 forms the interface with p62 near p44 contacts. G mutation would disrupt the H-bond network and p62 interaction, thereby resulting in TTD. However, in the NER complex, the p62 interaction is not present.
XPD	G675R	XP/CS	C/H	DOI: 10.1101/gad.859501. G675 lies at the end of a beta strand in a tightly packed core region. R mutation likely disrupts this tight hydrophobic core, reducing helicase activity (DOI: 10.1016/j.cell.2008.04.030), increasing rigidity and creating repulsion with nearby R616.
XPD	D681G/N/H	XP	C	DOI: 10.1101/gad.859501, 10.1002/humu.20768. D681 is N-capping at the end of a helix. It is also at the end of a beta strand and H-bonds to R683 (XP), S505, and S506. In the apo structure, R683 contacts XPB, while in the NER structure, R683 contacts ssDNA while also in a salt bridge with D681. G, N, or H mutation likely disrupts the helix and position of R683, and thereby coordination of helicase activities for NER.
XPD	R683W/Q	XP	C	DOI: 10.1101/gad.859501, 10.1002/humu.20768, 10.1073/pnas.94.16.8658. R683 lies on a loop between a strand and helix and contacts ssDNA. R to W mutation reduces ATPase activity, helicase activity and DNA binding (DOI: 10.1016/j.cell.2008.04.030). W or Q mutation likely disrupts interaction interface and coordination of helicase activities for NER, explaining disease phenotypes.
XPD	G713R	TTD	C/E<br>C/H	DOI: 10.1101/gad.859501. G713 is in the middle of a helix and faces a tightly packed hydrophobic core. This helix bridges between XPB and p44 in the apo and NER structures. In the PIC, the helix only interacts with p44. Mutation to R would lead to steric clashes with MET493 and ILE495.
XPD	A717G	XP	C/H<br>C/E	DOI: 10.1038/jhg.2015.18. A717 is right below G713 in the same helix and its methyl group goes into the same hydrophobic core formed by V530, V714, L721, L676, M493, and M527. In the NER complex, A717 is on a newly formed interface with XPB RecA2 (comm H), therefore likely involved in XPD regulation of XPB dynamics. G mutation next to G713 and R722 (XP) likely makes the helix overly flexible, uncoupling signaling with XPB.
XPD	R722W	TTD or XP/TTD	C/H	DOI: 10.1101/gad.859501, 10.1002/humu.20768. In two compound heterozygote patients with different disease phenotypes, the R722W mutation is found with

				S51F (XP/TTD phenotype) and with R378H (TTD phenotype). R722 is in the middle of a helix that bridges between p44 and XPB. The interaction of the helix with XPB may be involved in XPD inhibition and coordination of XPB helicase activity, similar to A717. Loss of salt bridge with D75 of p44 explains the TTD phenotype.
XPD	A725P/T/V	TTD	C/H	DOI: 10.1101/gad.859501, 10.4161/rdis.24932. A725 is at the end of a helix at the p44 interface. The main chain of A725 is interacting with E204 of p44. Mutation to P would lose this main chain interaction, while T and V as beta branched residues would sterically stick out against V221 of p44 and block the interaction.

*Disease abbreviations: Xeroderma pigmentosum (XP), trichothiodystrophy (TTD), Xeroderma pigmentosum/Cockayne syndrome (XP/CS)
